# αV-Integrins Are Required for Mechanotransduction in MDCK Epithelial Cells

**DOI:** 10.1371/journal.pone.0071485

**Published:** 2013-08-19

**Authors:** Terhi P. Teräväinen, Satu M. Myllymäki, Jens Friedrichs, Nico Strohmeyer, Jose V. Moyano, Chuanyue Wu, Karl S. Matlin, Daniel J. Muller, Aki Manninen

**Affiliations:** 1 Biocenter Oulu, Oulu Center for Cell-Matrix Research, Department of Medical Biochemistry and Molecular Biology, University of Oulu, Oulu, Finland; 2 Leibniz Institute of Polymer Research Dresden, Institute of Biofunctional Polymer Materials, Dresden, Germany; 3 ETH Zürich, Department of Biosystems Science and Engineering, Basel, Switzerland; 4 Biotechnology Center, Dresden University of Technology, Dresden, Germany; 5 Department of Pathology, University of Pittsburgh School of Medicine, Pittsburgh, Pennsylvania, United States of America; 6 Department of Surgery, Committee on Cell Physiology, and Committee on Molecular Pathogenesis and Molecular Medicine, The University of Chicago, Chicago, Illinois, United States of America; University of California, San Diego, United States of America

## Abstract

The properties of epithelial cells within tissues are regulated by their immediate microenvironment, which consists of neighboring cells and the extracellular matrix (ECM). Integrin heterodimers orchestrate dynamic assembly and disassembly of cell-ECM connections and thereby convey biochemical and mechanical information from the ECM into cells. However, the specific contributions and functional hierarchy between different integrin heterodimers in the regulation of focal adhesion dynamics in epithelial cells are incompletely understood. Here, we have studied the functions of RGD-binding αV-integrins in a Madin Darby Canine Kidney (MDCK) cell model and found that αV-integrins regulate the maturation of focal adhesions (FAs) and cell spreading. αV-integrin-deficient MDCK cells bound collagen I (Col I) substrate via α2β1-integrins but failed to efficiently recruit FA components such as talin, focal adhesion kinase (FAK), vinculin and integrin-linked kinase (ILK). The apparent inability to mature α2β1-integrin-mediated FAs and link them to cellular actin cytoskeleton led to disrupted mechanotransduction in αV-integrin deficient cells seeded onto Col I substrate.

## Introduction

The immediate cellular microenvironment, including the surrounding ECM, regulates cellular properties in different locations within tissues. Dynamic regulation of epithelial cell differentiation is crucial for tissue development and function. Epithelial cells are commonly underlined by a special type of ECM, the basement membrane (BM), which consists of laminins, collagen IV and various proteoglycans. In addition to the biochemical diversity of the ECM and its embedded signals (such as ECM-bound growth factors), mechanical properties of the ECM have a major influence on cellular responses [1]–[3]. Whereas laminin establishes a basal cue to guide apico-basal cell polarization [2], [4]–[7] the mechanical properties of the ECM are largely dependent upon fibrillar collagen and fibronectin (FN) networks. Interactions between epithelial cells and the ECM play an important role in the regulation of proliferation, survival and migration of normal and carcinoma cells [8], [9].

Dynamic assembly and disassembly of integrin-mediated focal adhesions is crucial for epithelial cell migration, mechanotransduction and epithelial morphogenesis. Integrins are heterodimeric cellular receptors for laminins, collagens and FN and have been reported to actively participate to the hierarchical co-assembly of these interconnected networks [10]–[14]. Integrins are also crucial components of the tension-sensing machinery that detects the mechanical properties of the ECM [2], [15]. Most cells express many different integrin heterodimers that interact with partially overlapping repertoires of ECM molecules. A complex signaling cross-talk operates between the different integrin species [16]–[19]. While β1- and β4-integrins appear to mediate collagen adhesion and the laminin-based basal cue guiding epithelial cell polarity [5], [6], [20], the functional roles of a promiscuous group of RGD-motif binding integrins in epithelial cells are less clear. RGD-motifs are abundant in both ECM proteins and soluble factors [21]. In fibroblasts adhering to FN, αV- and β1-integrins cooperate to form focal adhesions [2], [15]. However, the specific role of RGD-binding αV-integrins in epithelial cell adhesion or their possible functional interplay with β1-integrins is not thoroughly understood.

Here, we have studied the functional roles of RGD-binding integrins expressed in epithelial Madin Darby Canine Kidney (MDCK) cells and their possible crosstalk with β1-integrin-dependent functions. Interestingly, αV-integrins were found to regulate cell spreading not only on FN but also on other ECM substrates such as collagen I (Col I) and LN-511 to which adhesion was mediated by β1-integrins. The surface exposure or initial binding of α2β1-integrins (the main collagen receptor in MDCK cells) to Col I was not affected in αV-integrin knockdown (ItgαV-KD) MDCK cells, but the recruitment of talin and multiple other components of focal adhesions (FAs) was abrogated resulting in perturbed mechanosensory responses. Whereas inhibition of talin-1, FAK or ILK expression led to impaired cell spreading only depletion of talin-1 replicated the defect in cellular mechanotransduction seen in ItgαV-KD cells. These findings identify a novel role for αV-integrins in modulating talin-dependent mechanotransduction in epithelial MDCK cells.

## Results

### Characterization of the functional roles of RGD-binding integrins in MDCK cells

To study the functions of RGD-binding integrins in epithelial cells we analyzed the integrin mRNA expression profile in non-transformed MDCK cells by quantitative PCR. Out of the RGD-interacting integrin subunits, MDCK cells expressed β1-, β3-, β5-, β6-, β8-, α5- and αV-integrins (data not shown, see also [6]). To study the functional roles of these integrin subunits, we depleted their expression in MDCK cells using RNA interference (RNAi). Efficient depletion of the target mRNAs was confirmed using qPCR (**Table S1A**). The depletion of the different integrins at protein level was analyzed using western blotting and metabolic labeling experiments (**Fig. S1**).

FN can serve as a ligand for all of the above-mentioned integrins [22]. Moreover, α5β1- and αVβ3-integrins are also central for the assembly of FN matrices in some cell types [23]. Adhesive properties of the different Itg-KD cells on FN were analyzed employing a cell washing assay [24]. These data indicated that αVβ6-integrin is the main adhesion receptor for FN in MDCK cells (**Fig. S2A**). β1-integrins were crucial for efficient adhesion to collagen I (Col I), laminin-511 (LN-511) and basement membrane extract (BME) but they were dispensable for FN adhesion (**Fig. S2**, see also [6]). Surprisingly, in addition to Itgβ1-KD cells, a notable proportion of ItgαV-KD cells detached during washing steps from Col I-, LN-511- and BME-coated wells (**Fig. S2B–D**).

**Figure 1:**
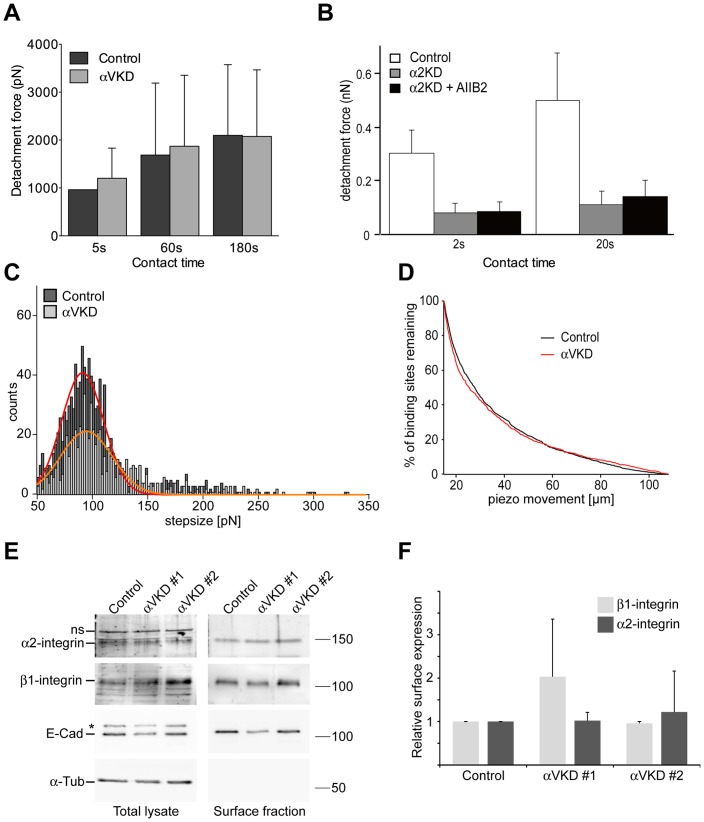
αV-integrins do not contribute to early adhesion to collagen I in MDCK cells. **A**) Quantifying early cell adhesion by SCFS. Single control or ItgαV-KD cells were captured onto ConA-coated AFM cantilevers and pressed onto Col I-coated substrates. After the indicated contact times, cells were lifted from the substrate, and the maximum detachment force was recorded as described in materials and methods. More than 100 F–D curves were recorded for each condition. Mean+SD is shown. **B**) The maximum detachment force between collagen I substrate and control or Itgα2-KD MDCK cells was measured in the absence and presence of antibodies that block β1-integrin adhesion (AIIB2). At least 20 cells were analyzed for each condition. Values show mean ± SD. Membrane tether analysis (see T in **Fig. S3A**): **C**) The distributions of tether rupture forces for control and ItgαV-KD cells both center at a value of ≈92 pN, indicating no differences in cell membrane properties. 78 F–D curves for control and 41 F–D curves for ItgαV-KD cells were analyzed. **D**) Membrane tether length analysis of control and ItgαV-KD cells to identify possible differences of receptor affinity and cell membrane-cortex interactions. A very similar tether length distribution does not indicate any changes. To assure that analyzed rupture events exclusively originated from membrane tethers force steps within 7 µm after the maximum detachment force were omitted from analysis. **E**) Control and two independent ItgαV-KD MDCK cell lines were allowed to spread on Col I-coated plastic tissue culture dishes for 75 minutes followed by surface biotinylation. After cell lysis, biotin-bound molecules were precipitated with avidin-beads and relative amounts of β1-and α2-integrins in biotinylated vs. total lysates were determined by SDS-PAGE and western blotting as described in materials and methods. E-Cadherin (E-Cad, asterisk indicates an intracellular precursor of E-Cad) and β-actin were used as biotinylation and loading controls. ns  =  non-specific. **F**) Quantification of the relative surface expression of β1- and α2-integrins in the indicated cell lines.

Our earlier data implicated α2β1-integrin as an important Col I receptor in MDCK cells [6]. However, because laminins and collagens also have RGD domains [25]–[28], we next tested whether αV-integrins might directly interact with Col I. The cell washing assay provides only semi-quantitative data about relative resistance of cells to shear forces during washing steps. To quantitatively compare the adhesion forces of control and ItgαV-KD cells to Col I substrate, an atomic force microscopy (AFM)-based single cell force spectroscopy (SCFS) assay was employed [29], [30]. We found that initial adhesion strengths of control and ItgαV-KD cells to Col I were similar (**Fig. 1A**). SCFS-measurements confirmed that Itgα2-KD cells have very poor Col I binding capacity indicating that adhesion to Col I is mediated via α2β1-integrins (**Fig. 1B**). Therefore, it is unlikely that αV-integrins directly contribute to Col I binding. Analysis of membrane tethers (see **Fig. S3A**), formed upon separating control and ItgαV-KD cells from Col I substrate, did not reveal significant differences in the tether rupture force (**Fig. 1C**) or in the number or length of tethers (**Fig. 1D**). As the tether rupture force and the length of membrane tethers depend both on the membrane properties and lifetime of receptor-ligand bonds [31] it can be also assumed that the availability of α2β1-integrins at the cell surface, their affinity to Col I and the membrane-cortex interactions were similar in control and ItgαV-KD cells.

Given the important role of α2β1-integrin in adhesion to collagen the availability of α2β1-integrins at the cell surface was also confirmed by a surface biotinylation assay. The surface biotinylation assay did not reveal significant differences in the surface exposure of α2- or β1-integrin subunits (**Fig. 1E–F**). Moreover, metabolic labeling experiments showed that the steady-state expression levels and the composition of β1-integrin heterodimers were similar in control and in ItgαV-KD cells where αV-integrin protein levels were undetectable (**Fig. S1G**). Incubation of MDCK cells with short RGD-motif containing polypeptides efficiently inhibited FN adhesion but did not perturb adhesion to LN-511 or Col I (unpublished observations, see also [24], [32], [33]). Finally, in MDCK cells seeded onto FN, αV-integrin-red fluorescent protein (RFP) fusion protein accumulated to pericellular foci whereas no such foci were seen on Col I substrate where relatively uniform membrane staining was observed (**Fig. S1H**). Thus, we conclude that αV-integrins do not play a significant role as collagen binding receptors in MDCK cells which instead adhere to collagen mainly via α2β1-integrins.

### αV-integrins regulate cell spreading on collagen I and laminin-511 matrices

**Figure 2:**
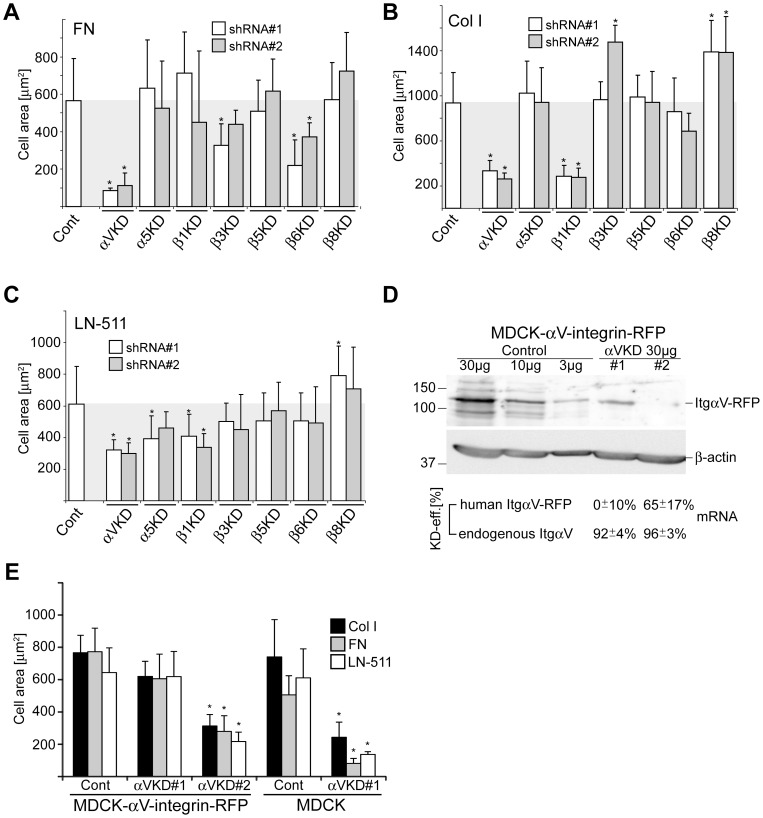
αV-integrins are required for efficient cell spreading on multiple ECM substrates. Control and the indicated Itg-KD MDCK cells were plated on **A**) fibronectin-, **B**) Col I- or **C**) LN-511-coated glass coverslips and allowed to spread for 75 minutes. Cells were fixed and filamentous actin was stained using TRITC-Phalloidin. The combined data shows the mean cell spreading area (µm^2^/cell) +SD of 3–6 independent experiments. For each experiment and coating condition 60–150 cells from 10–30 frames were analyzed. **D**) MDCK cells stably expressing human αV-integrin-RFP fusion protein were transduced with control, ItgαV-KD#1 or ItgαV-KD#2 shRNA-expressing viral vectors. The indicated amounts of total cell lysates were loaded onto SDS-PAGE followed by detection of human αV-integrins by western blotting (upper panel). Actin was used as a loading control (lower panel). KD-efficiencies for both endogenous and ectopically expressed αV-integrins were also determined using qPCR. Note that human αV-integrin is resistant to silencing by shRNA#1 but not shRNA#2 whereas both shRNAs efficiently silence the expression of canine αV-integrin (see also Fig. S1). **E**) The spreading of the different MDCK-αV-integrin-RFP cell lines on Col I, FN and LN-511 was analyzed as above together with control and ItgαV-KD#1 MDCK cells. The data shows the mean cell spreading area +SD from 2 independent experiments performed in triplicates. P-values <0.01 are signified by (*).

The functional regulation of integrins underlies cell spreading dynamics that involves coordinated assembly and disassembly of integrin-mediated cell-matrix contacts, linkage of these contact sites to the cellular cytoskeleton leading to cell spreading coordinated by the actin network dynamics [34]. ItgαV-KD cells did not adhere to FN and only few poorly spread ItgαV-KD cells could be recovered on this substrate (**Fig. 2A**). In line with the observed FN adhesion defect, Itgβ6-KD cells spread poorly on this substrate (**Fig. 2A** **)**. Itgβ3-KD cells displayed a significant spreading defect on FN suggesting that it also contributes to the regulation of cellular response upon adhesion to FN (**Fig. 2A**). Robust spreading defects were not observed for any other Itg-KD cells on FN. A large proportion of Itgβ1-KD cells failed to attach on Col I and LN-511 substrates and those which survived washing steps showed impaired spreading (**Fig. 2B–C**). ItgαV-KD cells had a marked spreading defect on both Col I and LN-511, providing a likely explanation for their detachment in the cell washing assay. None of the potential αV-pairing β-subunit (β3, β5, β6 or β8) KDs showed significant cell spreading defects on Col I or LN-511 (**Fig. 2B–C**). Thus, depletion of multiple ItgαV-integrin containing heterodimers (ItgαV-KD cells) is required to impair cell spreading on these substrates.

To confirm that αV-integrin function underlies the observed cell spreading defect we transduced the MDCK cell line that stably expresses human αV-integrin-RFP with control and the two ItgαV-KD constructs to silence the expression of endogenous canine αV-integrin. The knockdown efficiencies were confirmed both at protein and mRNA level (**Fig. 2D**, see also Fig. S1A). ItgαV-shRNA#1-construct contains multiple mismatches with the human αV-integrin mRNA and it was ineffective in silencing the ectopically expressed human αV-integrin mRNA that consequently rescued cell spreading in ItgαV-shRNA#1 transduced cells on all of the studied substrates (**Fig. 2E**). On the contrary, ItgαV-shRNA#2 contains only one mismatch with the human αV-integrin sequence close to the 3′-end of the target sequence and it is expected to significantly reduce the expression of not only the endogenous canine αV-integrin but also the ectopically expressed human αV-integrin-RFP [35]. Indeed, ItgαV-shRNA#2 efficiently down-regulated the expression of human αV-integrin-RFP protein thereby leading to cell spreading defect on Col I, LN-511 and FN (**Fig. 2D–E**). We also tested the effect of αV-integrin depletion on the spreading of some other cell lines. Although the spreading of MK3 cells, a cell line derived from mouse embryonic kidney, was somewhat less pronounced on collagen substrate when compared with MDCK cells, a significant reduction in spreading of MK3 cells was seen upon depletion of αV-integrin expression (**Fig. S4**). In addition, αV-integrin depleted human umbilical vein endothelial cells displayed a spreading defect on Col I substrate (data not shown).

### αV-integrins regulate the dynamics of β1-integrin-mediated focal adhesions

**Figure 3:**
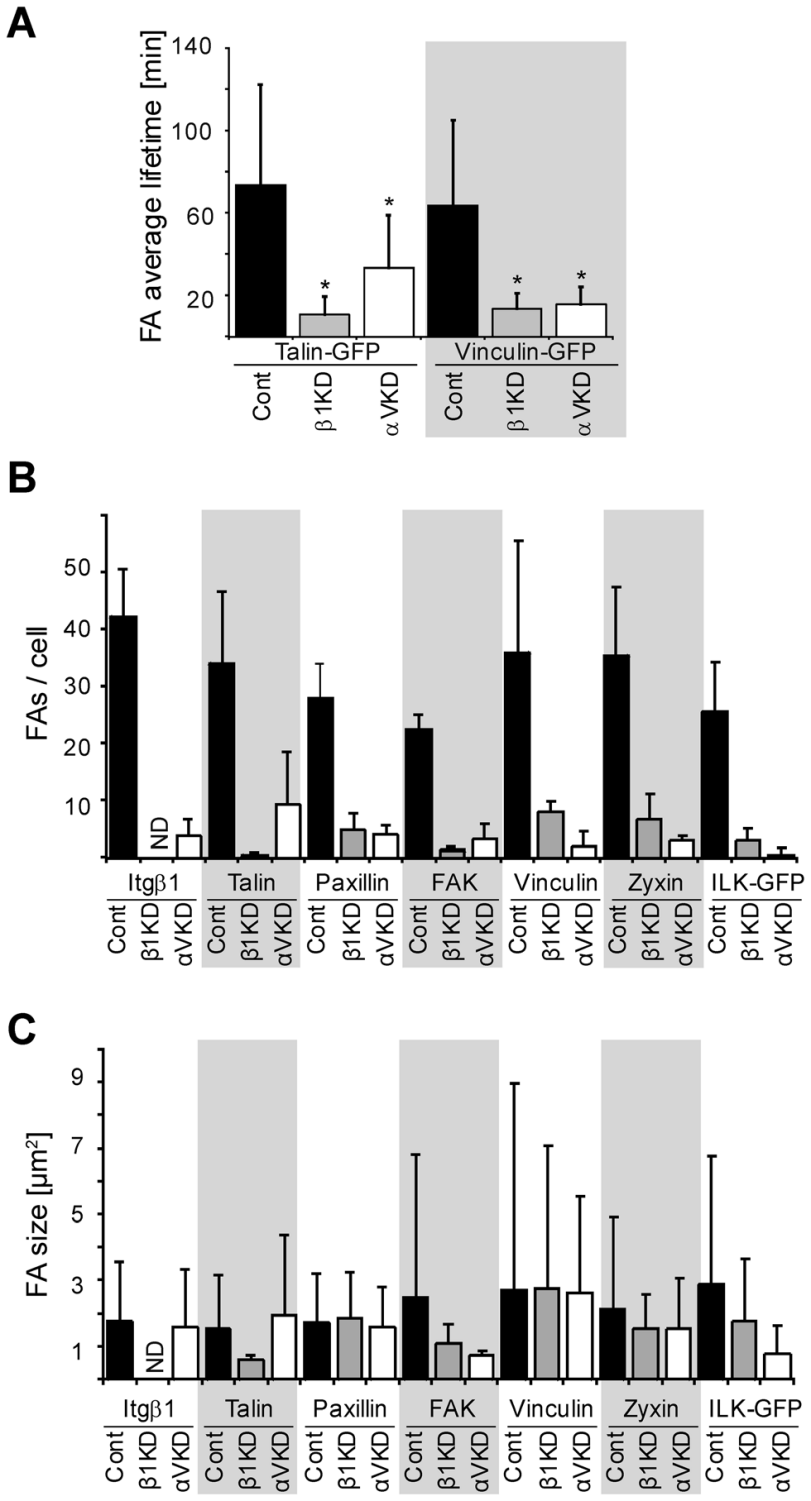
β1-integrin-mediated focal complexes form but do not mature on collagen I in the absence of αV-integrins. **A**) Control, Itgβ1- and ItgαV-KD MDCK cells stably expressing GFP-talin (Movie S2) or GFP-vinculin (Movie S3) were trypsinized and seeded onto Col I-coated glass-bottom petri dishes in serum-free culture medium. Cells were allowed to react with the substrate for 30–60 minutes after which the dynamics of FAs were imaged using timelapse microscopy. Images were acquired every 3 minutes and the average lifetime of FAs was determined as described in materials and methods. More than 50 FAs were tracked per each condition. P-values <0.01 are signified by (*). **B**) Control, Itgβ1- and ItgαV-KD MDCK cells were trypsinized, seeded onto Col I-coated coverslips, allowed to settle for 75 minutes, fixed and stained for the indicated FA markers. The number and **C**) the average size of FAs with each of these markers were analyzed as described in materials and methods. For each sample 5–15 cells were analyzed and the data show mean+SD combined from 3 independent experiments. ND: not detectable.

To study cell spreading dynamics in more detail we performed timelapse imaging of control, ItgαV- and Itgβ1-KD MDCK cells stably expressing GFP-talin or GFP-vinculin on Col I substrates. Talin binding to the cytoplasmic domain of β1-integrins is considered as an early event depicting integrin activation leading to the formation of focal complexes (FXs) whereas accumulation of vinculin is thought to occur upon stabilization of focal adhesions (FAs) due to robust linkage to actin cytoskeleton [34], [36]. The half-life of GFP-positive pericellular foci was analyzed in the different Itg-KD cell lines. Upon initial contact, control cells protruded prominent lamellipodia which progressively stabilized leading to cell spreading and flattening of the cell body (**Movie S1**). Both GFP-talin and GFP-vinculin were efficiently recruited to peripheral foci most of which persisted for ∼60 minutes (**Fig. 3A**). In contrast, the lamellipodia in ItgαV-KD cells went through continuous protrusion-retraction cycles and showed reduced amount of GFP-talin positive foci that were more short-lived (<40 minutes; **Movie S2**) than in controls. The average residence time of GFP-vinculin in these foci was even shorter (10–20 minutes; **Movie S3**). Similar to ItgαV-KD cells, only short-lived dynamic GFP-vinculin- or GFP-talin-positive foci were visible in Itgβ1-KD cells (**Fig. 3A**). Curiously, unlike ItgαV-KD cells where the spreading phenotype persisted, overnight cultures of Itgβ1-KD cells eventually formed stable vinculin-positive adhesive structures and spread (data not shown). This is possibly due to secretion and assembly of endogenous ECM, such as LN-511 or FN that can be bound by other integrins and non-integrin receptors [37]. These data show that αV-integrins are required to stabilize α2β1-integrin-mediated FAs on Col I.

### αV-integrins are required for efficient maturation of FAs

**Figure 4:**
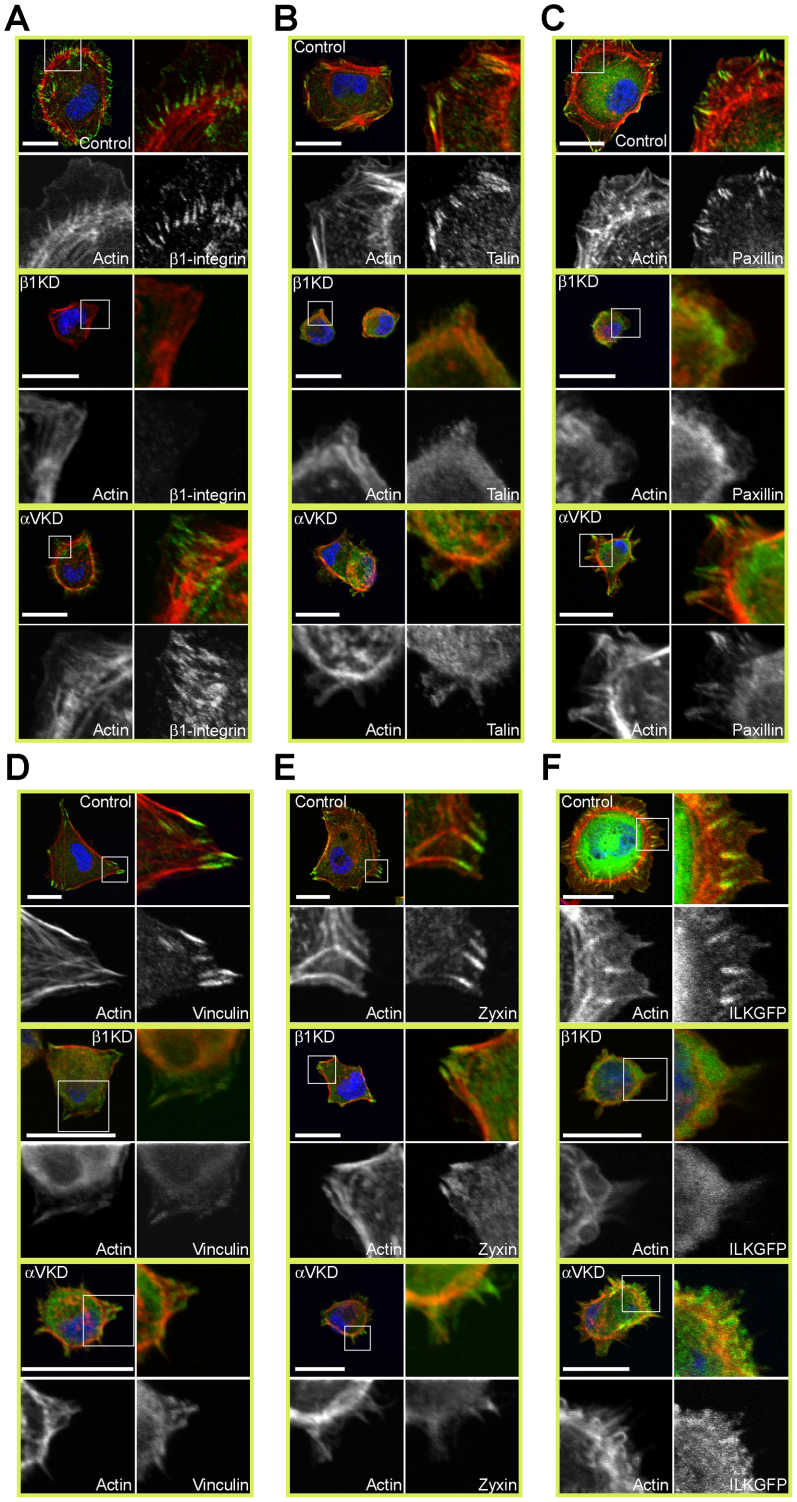
Recruitment of multiple FA components is defective in ItgαV- and Itgβ1-KD ItgV-KD cells. **A**) Control, Itgβ1- and ItgαV-KD MDCK cells were trypsinized, seeded onto Col I-coated coverslips in serum-free culture medium, allowed to settle for 75 minutes, fixed and stained for actin (TRITC-phalloidin, red), nuclei (DAPI, blue) and β1-integrin (green), **B**) talin, **C**) paxillin, **D**) vinculin or **E**) zyxin. **F**) Control, Itgβ1- and ItgαV-KD MDCK cells expressing GFP-ILK (green) were treated as above and stained for actin (TRITC-phalloidin, red) and nuclei (DAPI, blue). Scale bars are 20 µm in all figure panels.

The maturation of FXs to FAs and ultimately to fibrillar adhesions is a complex and highly orchestrated process [38]. We studied the recruitment of selected FA components to the maturing FXs by analyzing the number and average size of peripheral foci immunostained with these selected markers (**Fig. 3B, C**). Efficient clustering of β1-integrins at lamellipodia was observed in control cells (**Fig. 4A**). β1-integrin clusters were observed also in ItgαV-KD cells although they were significantly less in number than those in control cells (**Fig. 4A**). In agreement with the GFP-talin data, endogenous talin was recruited to clearly distinguishable foci at FAs in control cells whereas it was rarely seen to accumulate in adhesions in ItgαV-KD cells or Itgβ1-KD cells (**Fig. 4B**). Paxillin, another proximal β1-integrin-binding protein, was recruited to small peripheral foci that aligned with the ends of actin stress fibers in control cells but only few diffuse structures were seen in ItgαV- and Itgβ1-KD cells (**Fig. 4C**). In agreement with timelapse experiments, the number of vinculin-positive FAs was significantly reduced in both Itgβ1- and ItgαV-KD cells when compared with control cells (**Fig. 4D**). Thus it appears that ItgαV-KD cells adhere to Col I via α2β1-integrins but these early β1-integrin-positive foci do not properly mature into FAs.

Mechanical forces acting upon cytoskeleton and cell-matrix contacts play an important role in FA maturation [34]. Forces generated by actomyosin contractility recruit zyxin to FAs where it regulates actin polymerization [39], [40]. In control cells, zyxin was robustly recruited to maturing FAs and aligned with adjacent stress fibers (**Fig. 4E**). Unlike the elongated sharp-edged structures in control cells, zyxin stained diffuse clusters in some Itgβ1-KD cells or was cytoplasmic in others (**Fig. 4E**). In ItgαV-KD cells zyxin did not show any evident clustering (**Fig. 4E**). Integrin-linked kinase (ILK), together with its binding partners PINCH1 and α-parvin, is thought to strengthen the interactions between integrins and F-actin thereby preventing disruption of the ECM-cytoskeleton linkage under mechanical stress [41], [42]. We generated MDCK cells stably expressing an GFP-ILK fusion protein [43]. GFP-ILK accumulated at FA and aligned with actin stress fibers in control cells (**Fig. 4F**). In contrast, in Itgβ1- and ItgαV-KD cells GFP-ILK recruitment to FAs was severely affected (**Fig. 4F**). Defective FA-recruitment of these factors in ItgαV-KD cells suggests that the linkage between the ECM-bound β1-integrins and the actin cytoskeleton is impaired.

**Figure 5:**
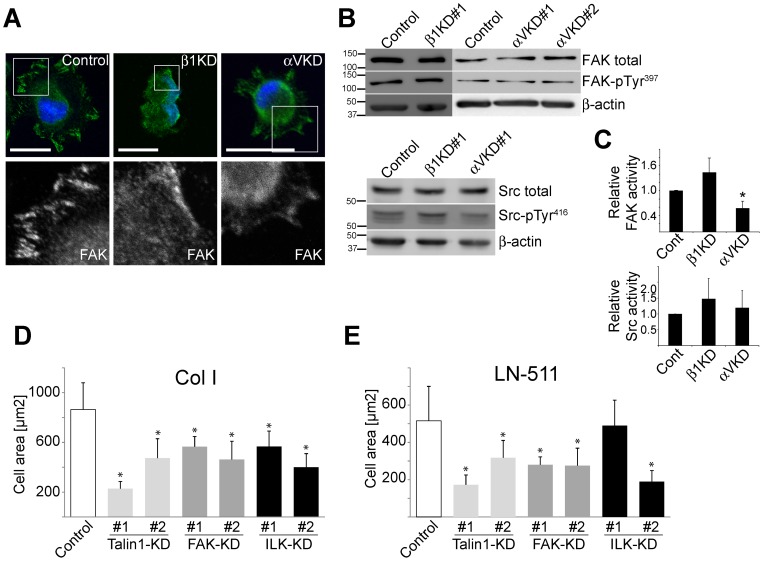
αV-integrins are required for proper FAK activation that together with talin-1 and ILK regulates MDCK cell spreading on Col I. **A**) Control, Itgβ1- and ItgαV-KD MDCK cells were trypsinized, seeded onto Col I-coated coverslips in serum-free culture medium, allowed to settle for 75 minutes, fixed and stained for actin (TRITC-phalloidin, red), nuclei (DAPI, blue) and FAK (green). Scale bars are 20 µm. **B**) Control, Itgβ1- and ItgαV-KD MDCK cells were trypsinized and seeded onto Col I-coated culture dishes. After 75 minutes cells were lysed and post-nuclear lysates were loaded onto SDS-PAGE followed by detection of total FAK and phosphorylated (pTyr^397^) FAK protein levels as well as total c-Src and phosphorylated (pTyr^416^) c-Src protein levels in the three cell lines. **C**) Quantitation of the FAK and c-Src activation in control, Itgβ1- and ItgαV-KD cells. The intensity ratios of FAK-pTyr^397^/FAK and c-Src-pTyr^416^/c-Src were quantified as described in material and methods from 3 independent experiments. Data is shown as mean+SD. **D**) Control, talin-1, FAK- and ILK-KD MDCK cells were plated on Col I- or **E**) LN-511-coated glass coverslips and allowed to spread for 75 minutes. Cells were fixed and filamentous actin was stained using TRITC-Phalloidin. The combined data shows the mean cell spreading area (µm^2^/cell)+SD of 3 independent experiments. For each experiment and coating condition 60–150 cells from 10–30 frames were analyzed. P-values <0.01 are signified by (*).

Talin and paxillin are thought to contribute to the recruitment of focal adhesion kinase (FAK) to regulate the maturation of FAs [44] whereas ILK interacts with the cytoplasmic tails of β1- and β3-integrins to link FAs to actin cytoskeleton and thereby promote FA maturation [45], [46]. FAK was efficiently recruited to FAs in control MDCK cells whereas limited a number of poorly organized FAK-positive foci were observed in Itgβ1-KD cells and only diffuse lamellipodial FAK-staining in ItgαV-KD cells (**Fig. 3B, C** **and** **5A**). Integrin clustering induces phosphorylation of FAK on the pTyr^397^-residue which provides a docking site for cellular proto-oncogene tyrosine-protein kinase (c-Src) and facilitates its activation (pTyr^418^) [47]. The resulting FAK/c-Src dual kinase complex activates multiple signals downstream of integrin activation [48]. Surprisingly, despite the different appearance of the FAK-localization in the Itgβ1-KD cells the depletion of β1-integrin did not reduce specific FAK or c-Src activation and instead a tendency for slightly elevated activity was noted in cells analyzed 75 minutes after seeding when compared with control cells (**Fig. 5B, C**). Despite normal c-Src-activation, ItgαV-KD cells failed to efficiently activate FAK upon adhesion to Col I (**Fig. 5B, C**).

To study the relative contribution of selected key components in FA maturation we generated talin-1 (Tln1)-, FAK- and ILK-KD MDCK cells and analyzed their spreading on Col I and LN-511 substrates. The KD-efficiencies were determined by qPCR (Tln-1, FAK and ILK; **Table S1A**). All of these KDs inhibited cell spreading on Col I substrate (**Fig. 5D**) and all but one of the two ILK-KD constructs impaired cell spreading on LN-511 (**Fig. 5E**). These data show that the failure of ItgαV-KD cells to recruit talin, FAK, and ILK to the forming adhesion on Col I substrate could underlie the cell spreading phenotype.

### Cellular mechanotransduction processes are defective in ItgαV-KD MDCK cells

**Figure 6:**
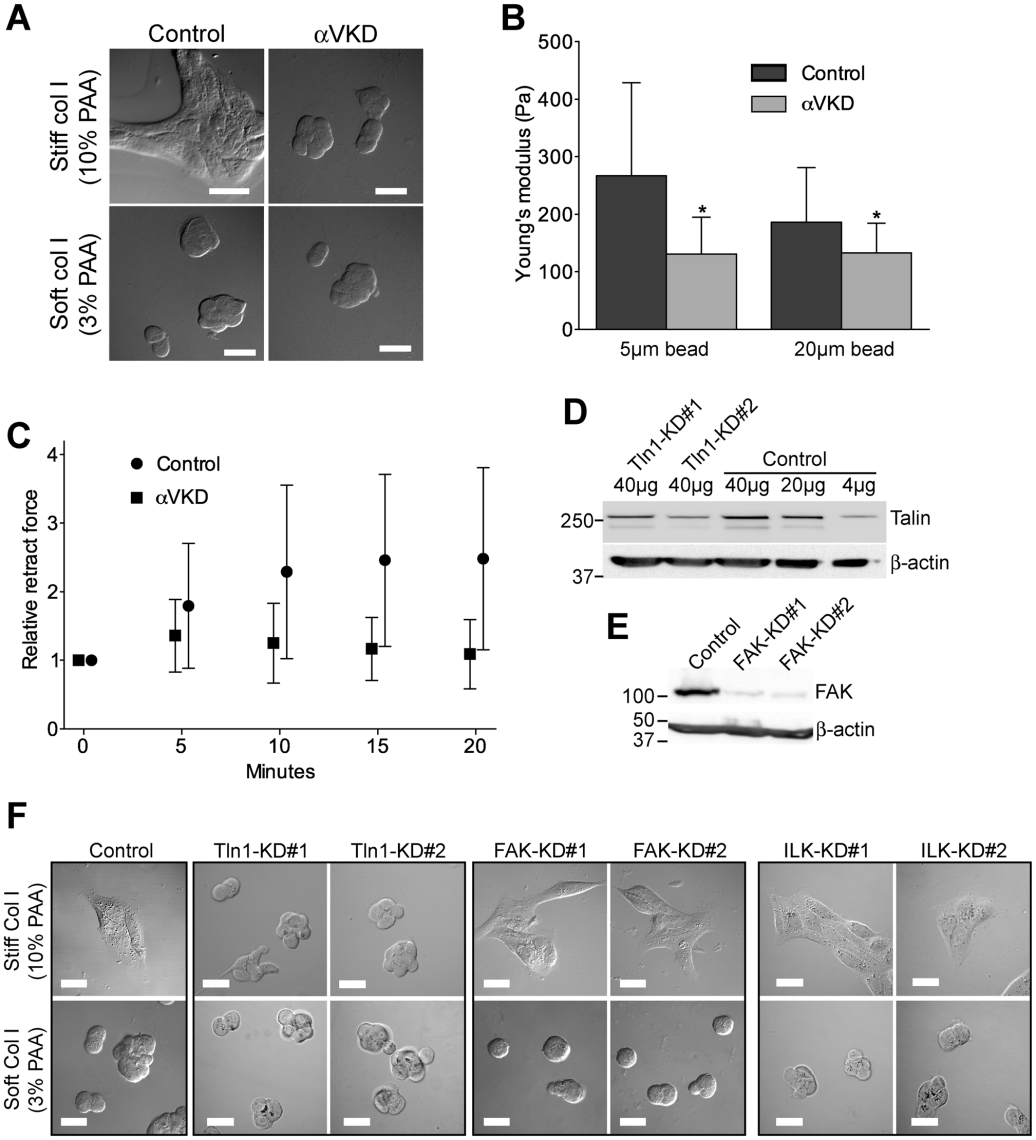
Mechanotransduction processes are abrogated in ItgαV-KD MDCK cells. A) Control and ItgαV-KD MDCK cells were trypsinized and seeded onto stiff (10% PAA) or soft (3% PAA) Col I-coated substrates. Cells were allowed to spread overnight followed by PFA-fixation and imaged using DIC-microscopy. Scale bars are 20 µm. B) Determining the cell elasticity of control and ItgαV-KD cells. Cells were seeded onto Col I-coated substrate and allowed to bind and spread for 12 hours. Cell elasticity was probed with 5 or 20 µm glass beads attached to an AFM cantilever. More than 60 cells per cell type and bead size were probed. Mean+SD is shown. P-values <0.01 are signified by (*). C) Control and ItgαV-KD MDCK cells growing on Col I-coated substrate were probed with a 5 µm bead attached to a tipless cantilever (Figure S3B). After making initial contact between Col I-coated bead and cell with a contact force of 1 nN for 120 seconds, the cantilever was oscillated at a frequency of 0.25 Hz and amplitude of 200 nm for 20 minutes. During oscillation, the force acting on the cantilever was recorded. More than 20 control and ItgαV-KD cells were analyzed. D) The indicated amounts of control and two independent talin (Tln)-1-KD cell line lysates were loaded for SDS-PAGE followed by western blotting with mouse anti-talin antibodies. E) Thirty micrograms of total lysates from control and two independent FAK-KD cell lines were loaded for SDS-PAGE followed by western blotting with mouse anti-FAK antibodies. F) Control and the indicated KD cell lines were trypsinized and seeded onto stiff (10% PAA) or soft (3% PAA) Col I-coated substrates. Cells were allowed to spread overnight followed by PFA-fixation and imaged using DIC-microscopy. Scale bars are 20 µm.

Integrin-mediated FAs function as cellular sensors to monitor the rigidity of the extracellular environment [34]. Because αV-integrins were found to be involved in the maturation of FAs their potential role in mechanotransduction was studied. To modulate substrate rigidity in vitro we used stiff (1893±394 Pa; 10% PAA) and soft (212±9 Pa; 3% PAA) polyacrylamide (PAA) gels that were coated with the same concentration of Col I [49]. The different cell lines were incubated on the substrates overnight. Control cells spread efficiently when allowed to settle on stiff Col I-coated gels whereas on soft substrates they did not flatten (**Fig. 6A**). On the other hand, ItgαV-KD cells had roundish “soft” morphology irrespective of the substrate rigidity (**Fig. 6A**). Janmey et al. reported that cells re-organize the actin cytoskeleton to increase their elastic properties in response to increasing substrate stiffness [50]. Thus, if the mechanotransduction machinery in ItgαV-KD cells is defective these cells would be softer than control cells on a rigid surface. Control and ItgαV-KD cells were seeded onto a stiff Col I-coated glass coverslip and allowed to adhere for 12 hours. Upon prolonged exposure on stiff surface, some ItgαV-KD cells flattened transiently. Quantitative cell elasticity measurements on such flattened ItgαV-KD cells in comparison with control cells were performed using AFM (**Fig. 6B**). Control cells had a rather broad spectrum of elasticities ranging from 100 to 1000 Pa (Young's modulus) with a mean value of 267 Pa when probed with 5 µm beads and 186 Pa when using larger 20 µm beads. ItgαV-KD cells were significantly softer with mean values of 130 Pa regardless of the bead size used, indicating that they did not respond to the substrate stiffness.

To directly measure the maturation of FAs upon mechanical stimulus we probed control and ItgαV-KD cells with a Col I-coated bead attached to an AFM cantilever (**Fig. S3B**). Cells were allowed to bind the bead for 2 minutes. Then, the bead was oscillated at a frequency of 0.25 Hz and an amplitude of 200 nm for 20 minutes. During oscillation, the bead was retracted to stress the attached cell and the force acting on the cantilever was measured. Repeated pulling of the bead consistently increased the mechanical response of the stressed control cells whereas the mechanical force response of ItgαV-KD cells decreased (**Fig. 6C**). The increasing mechanical resistance to bead movement in control cells likely represents the strengthening of the linkage between the FAs forming on the bead and the cellular actin cytoskeleton [15].

To dissect further which of the FA components contribute to mechanosensory responses we seeded talin-1-, FAK- and ILK-KD cells onto soft and stiff collagen-coated substrates and allowed them to grow on the substrates overnight. The depletion of talin-1 and FAK proteins were confirmed by western blotting (**Fig. 6D, E**). Whereas both talin-1-KD constructs shared the mechanotransduction defect seen with ItgαV-KD cells, FAK- and ILK-KD cells retained their capacity to spread on stiff Col I-coated substrate after overnight incubation implicating talin-1 as a critical component of the mechanosensory machinery in MDCK cells (**Fig. 6F**).

## Discussion

Functional characterization of RGD-motif binding integrins in MDCK cells revealed that αVβ6-integrin is the main adhesion receptor for RGD-containing FN while both αVβ6 and αVβ3-integrins contributed to cell spreading on FN substrate. Importantly, we observed that depletion of αV-integrins led to a marked cell spreading defect also on Col I and LN-511. Although αV-integrins reportedly can bind modified or denatured collagen [51] we did not observe significant αV-integrin-mediated binding to Col I in our experimental setups. Moreover, αV-integrins were not essential for the cell surface expression of α2β1-integrins which mediated Col I binding both in the presence and absence of αV-integrins. However, recruitment of talin, vinculin, ILK and zyxin to the forming adhesions as well as activation of FAK were abrogated in ItgαV-KD cells. Consequently, β1-integrin-mediated adhesions on Col I substrate did not properly link to cellular cytoskeleton leading to defective FA maturation and impaired mechanosensory responses. We showed that expression of talin-1, but not FAK or ILK, was required for efficient mechanosensory responses in MDCK cells.

Our data reveals a presumably indirect functional cooperation between α2β1- and αV-integrins during MDCK cell spreading. Here, several scenarios are possible. One of the best understood examples of integrin cross-regulation is between αVβ3- and α5β1-integrins, both of which bind to FN. Ligation and rapid recycling of αVβ3 slows down surface transport of α5β1 from the perinuclear recycling compartment thereby promoting persistent migration of fibroblasts [17]. Inhibition of αVβ3-function results in increased surface exposure of α5β1-integrin which in turn facilitates random migration and invasiveness of fibroblasts and ovarian carcinoma cells [52]. We did not observe significant changes in the surface exposure of α2β1-integrin in ItgαV-KD cells. However, because the trafficking of β1-integrins was not addressed in detail in our study we cannot exclude that some β1-integrin recycling defects occur in ItgαV-KD cells.

An elegant recent study identified separate roles for α5β1- and αVβ3-integrins in fibroblasts adhering to FN. It was reported that α5β1-integrins determine adhesion strength whereas αVβ3-integrins enable mechanotransduction [15]. Both α5β1- and αVβ3-integrins were required for proper maturation of FXs into FAs. After initial co-localization at FX, α5β1-integrin gradually accumulated and formed more stable mature FAs behind lamellipodia while αVβ3 remained at the leading lamella continuously probing the ECM. Our data implicating αV-integrins as regulators of mechanical FA maturation while β1-integrins mediate substrate binding appears compatible with these findings. Some earlier studies using modified fibroblast cell lines have reported that both αVβ3- and α2β1-integrins can contribute to mechanical deformation of collagen gels independently of each other [53], [54]. However, in these studies αVβ3-integrins were reported to bind to Col I substrate whereas no significant αV-integrin-mediated binding to Col I was observed in our experiments. Roca-Cusachs et al. reported that talin did not contribute to the adhesion strength but was required for mechanotransduction [15]. Curiously, although both ItgαV- and Itgβ1-KD cells failed to recruit talin at FAs only ItgαV-KD cells displayed a mechanotransduction defect. A recent study reported that whereas talin-depleted mammary epithelial cells failed to recruit multiple FA components, such as vinculin and FAK, cell spreading was not compromised as the necessary linkage between FAs and the actin cytoskeleton might have been mediated by other molecules, such as tensin [55]. We observed that talin-1-KD MDCK cells failed to spread efficiently on stiff collagen substrate thereby suggesting that talin-1 is a critical component of the mechanosensory machinery in MDCK cells. It should be noted, however, that talin-1 is required for efficient integrin activation [56], [57]. Further studies are needed to determine whether the spreading defect in talin-1-KD MDCK cells results from a general inhibition of integrin activation or if talin-1 might also have a more specific role in cellular mechanotransduction. The differential dependence on talin-1 expression in the spreading of the two epithelial cell lines could be due to cell line specific effects but further studies are needed to clarify this discrepancy. Interestingly, in fibroblasts talin is not required for initial adhesion or formation of lamellipodial protrusions but is necessary for FA maturation and cell spreading [58].

Although αV-integrins were found to regulate recruitment of several FA components and modulate activation of FAK, our data implicated talin, but not FAK or ILK, as a crucial FA component that regulates mechanosensory responses in MDCK cells. Further studies are warranted to characterize the detailed molecular mechanisms how αV-integrins and talin-1 regulate maturation of β1-integrin mediated FAs in MDCK cells.

## Materials and Methods

### Antibodies and ECM proteins

Rat anti-β1-integrin (AIIB2, [59]), mouse anti-β1-integrin (TS2/16; [60]) and rabbit anti-β1-integrin [61] antibodies were kindly provided by Dr. K. Matlin (Department of Surgery, University of Chicago, Chicago, IL). Mouse anti-a2-integrin (5E8; [62]) was kindly provided by Dr. R.B. Bankert (Department of Microbiology and Immunology, University at Buffalo, Buffalo, NY). Rabbit-anti β1-integrin antibody was from Chemicon (ABI952). Mouse anti-αVβ3-integrin (LS-C15967), rabbit anti-β6-integrin (LS-C24779) and sheep anti-β5-integrin (LS-C36943) antibodies were from Lifespan Biosciences. Mouse anti-talin (#T3287, clone 8D4), rabbit anti-αV-integrin (#6617) and mouse anti-vinculin (#4505) were from Sigma. Rabbit anti-α2-integrin was from Santa Cruz (sc-9089). Mouse anti-paxillin (#AHO0492) was from Zymed. Mouse anti-FAK (#610088) was purchased from BD Transduction LaboratoriesTM and mouse anti-FAK (pTyr-397; #FM1211) from ECM Biosciences. Rabbit anti-Zyxin (ab1842) was purchased from Abcam. Cy2-, Cy3- and HRP-conjugated secondary antibodies were from Jackson Immunoresearch and Alexa488–conjugated secondary antibodies were from Invitrogen. TRITC-Phalloidin and DAPI were purchased from Sigma. c-Src-antibody Sampler Kit (#9935) was purchased from Cell Signaling Technology. Bovine dermal collagen I (PureColTM, Inamed), BME (MatrigelTM, BD biosciences) and placental laminin-511 (Sigma, L6274; [63]) were purchased.

### Cell culture and treatments

For 2D culture, MDCK cells were grown in minimum essential medium (MEM, Invitrogen) supplied with 5% fetal bovine serum (FBS) and 1% penicillin/streptomycin at +37°C in a humidified CO_2_-incubator. Mouse kidney mesenchyme (MK3) cells were grown in Dulbecco's modified Eagle's medium (DMEM, 4,5 g glucose/l, Invitrogen) supplemented with 5% FBS [64].

### Generation of knockdown and overexpression cell lines

Knockdown cells were created as described previously [65]. Two functional constructs (>70% KD efficiency as determined by qPCR) were generated for each gene. For the target sequences and oligos used for qPCR see Table S1. When indicated, individual integrin-KD shRNA-expressing MDCK cell clones were picked and expanded. GFP-Talin1 ([66], Addgene plasmid 26724), GFP-vinculin [67], GFP-ILK [43] and αV-integrin-TagRFP (Evrogen JSC, #FP361) expressing cell lines were generated by stable transfection using Amaxa nucleofection (Lonza GmbH), individual colonies were picked. When indicated, the resulting cell clones were subsequently transduced with the integrin-targeting shRNA constructs.

### Cell washing assay

Subconfluent cells were washed two times with PBS and once with PBS containing 2 mM EDTA/0.5 mM EGTA after which the cells were detached using MEM containing 4 mM EDTA/1 mM EGTA. Detached cells were centrifuged (1000rpm, 3 min, at RT) and resuspended at a density of 2×10^6^ cells/ml into MEM (w/o serum). 10^5^ cells per well were pipetted onto a 96-well plates and let to adhere for 90 minutes. Wells were coated with Col I (30 µg/ml), fibronectin (10 µg/ml), LN-511 (10 µg/ml) or BME (90 µg/ml) and blocked with 1% BSA as described [24]. Non-adherent cells were removed by washing four times with PBS containing 0.9 mM calcium and 0.49 mM magnesium (PBS^+^). Remaining adherent cells were fixed with methanol and stained with 0.1% crystal violet. Cells were lysed in 0.1% sodium deoxycholate/10 mM HEPES pH7 and absorption was measured at 544 nm.

### Cell spreading and FA assays

Cells were trypsinized and resuspended into serum-free MEM. 1.5–2×10^3^ cells were seeded onto 12Ømm coverslips and allowed to settle for 65–75 minutes. Coverslips were coated with Col I (3–30 µg/ml), fibronectin (10 µg/ml), LN-511 (5–10 µg/ml) or basement membrane extract (90 µg/ml) and blocked with 1% BSA prior to experiments. Specimens were washed with PBS^+^, fixed with 4% PFA in PBS^+^ and stained with DAPI and TRITC-phalloidin. Cells were imaged with an Olympus FV-1000 confocal microscope. The average cell areas in ten individual field-of-views (40xobjective) were determined using Image J [68].

For FA analysis the number and size distribution of FAs were determined using the ImageJ software. In short, background was subtracted using the rolling ball algorithm and the images were thresholded to highlight FA clusters. Cell centers were omitted from the analysis due to high background fluorescence and the outer regions of the cells were examined using particle analysis application to segment and measure the number and the average size of FAs. Only objects larger than 0.5 µm^2^ in size were included into the analysis. Five to fifteen cells per condition were analyzed. For analysis of FA dynamics the MJTrack plugin of the ImageJ was used to track randomly selected FAs in the timelapse series where images were captured every 3 minutes [69]. Between fifty-three to seventy-nine FAs were analyzed per each condition and the average FA lifetimes in minutes were determined.

### Single-cell force spectroscopy

SCFS was performed on an AFM (NanoWizard, JPK Instruments) equipped with a CellHesion module (JPK Instruments) mounted on an inverted optical microscope (Axiovert 200 M, Zeiss, Jena, Germany). Measurements were performed in media (MEM supplemented with 25 mM Hepes) at 37°C, controlled by a PetriDishHeater (JPK Instruments). Tipless, 200 µm long V-shaped cantilevers having nominal spring constant of 0.06 N/m (NP-O, Bruker) were calibrated using the equipartition theorem [70]. For SCFS, Ø35 mm glass-bottom petri dishes (WPI) were coated with Col I (PureCol™, Nutacon) as described [29]. AFM cantilevers were plasma-cleaned and coated with concanavalin A (ConA, Sigma, 2 mg/ml) overnight at 4°C. When indicated, rat anti-β1-integrin antibody (AIIB2–1∶10 dilution of hybridoma supernatant) was incubated with cells 20 minutes prior to SCFS experiments. To attach a single cell to the cantilever, cell suspensions were pipetted onto the collagen-coated supports. The apex of a ConA-functionalized cantilever was lowered at a velocity of 10 µm/s onto a cell until detecting a force of 1 nN. After a contact time of 5 s, the cantilever was withdrawn 90 µm and the cantilever-bound cell was left for incubation for >10 min. Then, the cantilever-bound cell was brought into contact with the collagen-coated support at a contact force of ≈2 nN for 5, 60 or 180 s. The approach and retract velocity of the cell was 5 µm/s. The deflection of the cantilever was recorded and plotted as force-distance (F–D) curves. After recording 25–35 F–D curves, the cell on the cantilever was replaced and after 40–60 F–D curves the support was exchanged. Each dataset was generated using 20–25 cells. The maximum detachment force required to separate cell and support was extracted from retraction F–D curve using the AFM data processing software (JPK Instruments). Tether rupture forces and tether lengths were extracted from retraction F–D curves using in-house algorithms in Igor Pro 6.12 (WaveMetrics, Oregon, USA). Unbinding events within 7 µm after the maximum detachment force were excluded from tether analysis.

### AFM-based nanoindentation experiments

The Young's moduli of cells and PAA gels were determined by AFM-based nanoindentation measurements. Before measurements, cells were seeded onto collagen-coated Ø35 mm glass-bottom petri dishes (WPI) and incubated for 12 hours. PAA gels were formed as surface bound layers with a final thickness (swollen state) of approx. 200 μm. After polymerization, each gel sample was washed in PBS to remove any non-bound polymeric precursors. Measurements were conducted at 37°C in PBS using a PetriDishHeater™ (JPK Instruments) sample chamber.

Colloidal force probes were prepared by attaching glass beads (Kisker Biotech) of various diameters (5 and 20 μm for cell measurements; 10 µm for PAA gel measurments) to the apex of tipless silicone nitride cantilever (NP-O, Bruker) using a two-component epoxy glue (Araldit) as described [70]. Spring constants of the colloidal probes were calibrated before measurements using the equipartition theorem [71]. To prevent non-specific adhesion, the modified cantilevers were incubated in heat inactivated FBS (Invitrogen) for 1 hour prior to measurements. For nanoindentation measurements, the approach and retract velocity was set to 1 µm/s, the contact force to 0.5 nN, and the pulling range was 2 µm. Experiments were performed in closed-loop, constant height mode. For cell measurements, up to three F–D curves, with at least 15 s waiting time between successive curves were recorded per cell. For PAA gel measurements, at least 100 different spots on 3 different samples were analysed. The data processing software provided by the AFM manufacturer (JPK Instruments) was used to extract the Young's Modulus E from approach force-distance curves. The software applies a modified Hertzian fit assuming a spherical indenter.

### Measuring mechanical maturation of FA using AFM

Cells were seeded onto collagen-coated Ø35 mm glass-bottom petri dishes (WPI) and incubated for 12 hours. Colloidal force probes were prepared by attaching a 5 µm glass bead to the apex of a tipless cantilever as described. Bead-modified cantilever were coated with Col I as described [29]. Measurements were performed in media (MEM supplemented with 5% FCS and 25 mM Hepes) at 37°C. For measurement of the mechanical maturation of FA, a bead-modified, functionalized and calibrated cantilever was lowered onto the margin of a single cell until a contact force of 1 nN was reached and was incubated in constant height mode for 2 minutes to ensure proper binding of the functionalized bead to the cell (**Fig. S3B**). Then, the bead-modified cantilever was repeatedly oscillated with amplitude of 200 nm and a frequency of 0.25 Hz for 20 minutes. During each oscillation cycle, a F–D curve was recorded. The maximum force of the retract F–D curve was extracted using the AFM data processing software (JPK Instruments). A relatively high variability in initial forces resisting the cantilever retraction (1–4 nN) were observed and thus the retract force of each cycle was normalized against the value of the first cycle. The normalized values from each analyzed cell were then averaged for the different cell types.

### Immunofluorescence

For immunofluorescence analyses, cells on top of glass coverslips or PAA-gels were fixed with 4% PFA in PBS^+^ for 10 min, washed with PBS^+^ and subsequently blocked and permeabilized by 20 minutes incubations in 200 mM glycine in PBS and 0.5% BSA, 0.2% gelatin in PBS with 0.1% TX-100. Samples were incubated overnight in +4°C with the indicated primary antibodies, washed four times with the blocking solution and incubated for 1 hour at RT with secondary antibodies. Samples were washed with PBS and mounted on object glasses or, in the case of PAA-gel cultures, on top of larger cover glass using ImmuMount^TM^ (Thermo Scientific).

### Timelapse microscopy

Timelapse imaging was done using a CSUX1M 500, Yokokawa CSU-X1 spinning disk unit equipped with a Hamamatsu EM-CCD camera (512×512) and mounted on an Axio Observer Z1 (Zeiss) microscope using either 25X LD LCI Plan-Apochromat Multi-immersion (NA0.8, WD 0.57 mm) or 63X Plan-Apochromat Oil immersion (NA1.46, WD 0.1 mm) objectives at +37°C in a humidified stage-top CO_2_-incubator (PeCon GmbH). Alternatively, a TIRF-setup assembled on the same microscope frame was used in combination with the above-mentioned 63x objective and Hamamatsu ORCAII CCD-camera. For timelapse imaging, cells were trypsinized, washed once with PBS, resuspended into serum-free MEM and seeded onto glass-bottom culture dishes (Cellview^TM^, Greiner BioOne) that had been coated with 3 µg/ml Col I, blocked using 1% BSA in PBS and washed twice with PBS and once with serum-free MEM prior to adding cell suspension.

### Cell culture on PAA gels

Cells were seeded onto collagen-coated polyacrylamide (PAA) gels of different elasticities. PAA gels were prepared with final concentration of acrylamide of 3 and 10% w/v from commercial 40% acrylamide/bisacrylamide solution (37.5∶1) (SigmaThe gels contained 54 mM Hepes pH 7.0 and 1/2000 vol TEMED and 0.05% APS. 20 µl or 40 µl of PAA gels was applied on top of Surfacil (Pierce)-coated object glass and an activated coverslip was placed on top. Acid washed (incubated for 16 hours in 1 M HCl at 55°C, rinsed with water, washed with 70% EtOH prior to o/n incubation in 100% EtOH), dried and autoclaved coverslips were activated with 0,5% 3-aminopropyltrimethoxysilane (Sigma) in H_2_O, washed thrice with H_2_O, incubated with 0.5% glutaraldehyde in H_2_O, rinsed again with water and air-dried. PAA gels were let to polymerize for 8 minutes and washed with water. The gels were activated with 1 mg/ml NHS/Nitrophenyl Azide Crosslinker Sulfo-SANPAH (#22589, Thermo Scientific) in H_2_O by 15 minutes incubation under UV light followed by washing with 200 mM HEPES pH8.5. Col I (150 µg/ml) was added in 20 mM Hepes pH 8.5 and incubated o/n +4°C. ECM-linked gels were washed several times with PBS prior to experiments. Different acrylamide concentrations were tested and the experimental conditions were selected such that in stiff (10%) PAA gels most of the control cells spread efficiently whereas very little spreading of ItgαV-KD cells was observed. In the very soft (3%) PAA essentially none of the cells showed any significant degree of spreading.

### Immunoblotting

Cells were lysed either directly in 1xSDS sample buffer (for c-Src; 100 mM Tris-HCl, pH 8.8, 2.5 mM EDTA, 10% sucrose, 0.1% bromophenol blue, 10 mM dithiothreitol, and 1% SDS) or FAK lysis buffer (9.1 mM Na2HPO4, 1.7 mM NaH2PO4 pH 7.2, 1% NP40, 0,25% sodium deoxycholate, 150 mM NaCl, 0,1% SDS, 1 mM EDTA) supplemented with protease (P8340 Sigma) and phosphatase (Sigma P5726) inhibitor cocktails. Proteins were separated using SDS-PAGE (Biorad) and transferred onto Protran nitrocellulose filters (Perkin-Elmer). Specific proteins were detected with indicated antibodies followed by chemiluminescence based on HRP-conjugated secondary antibodies (Pierce). Densitometric analysis was done using LAS-3000 imaging system (Fujifilm). The intensity ratios of activated (tyrosine-phosphorylated) FAK/c-Src to total FAK/c-Src were determined using Quantity One (Biorad). The ratio in control cells was set to 1 and the ratios from Itgβ1-KD and ItgαV-KD cells are shown relative to that.

### Surface biotinylation and metabolic labeling of integrins

The high pH method was used [72]. 3×10^6^ cells were plated onto collagen-coated and BSA-blocked Ø100 mm dishes as described for washing assays. After 75 minutes, cells were washed once with PBS^+^ and once with TEA-buffer (10 mM triethanolamine, 150 mM NaCl, 2 mM CaCl_2_, and 0.5 mM MgCl_2_, pH 9.0) followed by incubation with Sulfo-NHS-SS-biotin (0.5 mg/ml in TEA, #21331, Pierce) for 30 minutes on ice and washed twice with ice-cold PBS^+^. Cells were extracted with 0.5 ml of TNE-buffer (10 mM Tris-HCl pH 7.2, 2% Triton-X100, 150 mM NaCl, 1 mM EDTA) supplemented with protease and phosphatase inhibitor cocktail (#04693116001, Roche) and 10 mM glycine, scraped to microtubes, and inverted at 4°C for 30 minutes. Insoluble material was removed by centrifugation (15000×g, 4°C 10 minutes). Protein concentration of the supernatant was determined with the bicinchoninic acid assay (23227, Pierce). 4xsample buffer was added and samples were heated at 95°C for 3 minutes. Biotinylated proteins were precipitated (o/n at 4°C) with 30 µl of avidin-agarose beads (20219, Pierce) and released by heating (95°C, 3 minutes) in 30 µl of 2xsample buffer. Samples were separated by SDS-PAGE and immunoblotted as described above.

Metabolic labeling was done as described previously with minor adjustments [61]. Cells were detached and washed twice with long term labeling medium containing 1/10^th^ MEM and 9/10^th^ DMEM without Methionine/Cysteine/L-glutamine (#D0422, Sigma), supplemented with 5% FBS, L-alanyl-L-glutamine (#A12860-01, Invitrogen) and 1% penicillin/streptomycin. 4.4×10^4^ cells per cm^2^ were plated (Ø60 mm dishes) in long term labeling medium containing 100 µCi of EXPRE^35^S^35^S Protein Labeling Mix (#NEG072, Perkin Elmer) and cultured for 18 hours. Radiolabeled cells were washed three times with ice-cold PBS^+^ and extracted in 1 ml of RIPA-buffer (10 mM Tris-HCl pH 7.5, 0.5% SDS, 1% IGEPAL, 0.15 M NaCl, 1% Sodium deoxycholate + protease and phosphatase inhibitors; 4°C, 30 minutes). Insoluble material was removed by centrifugation (4°C, 10 minutes) and lysates were clarified by filtering through Ultrafree-CL centrifugal filter units (#UFC40HV00, Millipore). Protein concentration of the filtrate was determined with the bicinchoninic acid assay. Samples were incubated overnight with primary antibodies at 4°C and antigen-antibody complexes were recovered by adding 30 µl of Protein A-Trisacryl (#20338, Pierce) and inverting for further 2 hours. Immunoprecipitates were pelleted (500×g, 15 seconds) and washed thrice in RIPA-buffer and once with 10 mM Tris, pH 8.6. Trisacryl beads were resuspended in 2xsample buffer and heated at 95°C for 3–5 minutes. The samples were then alkylated with iodoacetamide for 20 minutes at 37°C and fractionated by SDS-PAGE. Gels were fixed, impregnated with EN3HANCE (Perkin Elmer), dried and exposed to KODAK Biomax XAR film (#1651454001EA, Perkin Elmer).

## Supporting Information

Figure S1 **Analysis of integrin protein expression in the different integrin-KD cell lines. A**) The indicated amounts of control and ItgαV-KD#1 and #2 MDCK cell lysates were loaded for SDS-PAGE followed by detection of αV-integrins by western blotting with rabbit anti-αV-integrin antibodies. β-actin was blotted as a loading control. **B**) Control MDCK and two independent ItgαV-KD cell lines were grown for 18 hours on Ø60 mm TC-plastic dishes and metabolically labeled for 18 hours with ^35^S-Methionine/Cysteine. αV-integrins were immunoprecipitated with rabbit polyclonal anti-αV-integrin antibodies as described in materials and methods. **C**) Control and Itgβ1-KD#2 MDCK cell lines were grown and metabolically labeled as in B) followed by immunoprecipitation of β1-integrins using rabbit polyclonal anti β1-integrin antibodies. The identity of the protein bands was confirmed with a series of metabolic labeling experiments using Itgα2- (Fig. S1G) and Itgα3-KD (data not shown) cells in which the respective protein bands were significantly reduced. **D**) Twenty micrograms of control and two Itgβ3-KD MDCK cell lysates were loaded for SDS-PAGE followed by detection with mouse monoclonal αVβ3-integrin antibodies. Only a faint band at ∼95 kDa was observed in the control cell lysate but the intensity of this band was further reduced in Itgβ3-KD#2 cells and it was undetectable in Itgβ3-KD#1 cell lysates **E**) The indicated amounts of control and two independent Itgαβ6-KD MDCK cell lysates were loaded for SDS-PAGE followed by detection of β6-integrins by western blotting with rabbit anti-β6-integrin antibodies. The antibody recognized two bands (∼110 kDa and ∼85 kDa) both of which appeared to be reduced in Itgβ6-KD cell lines. The calculated molecular weight of canine β6-integrin is 86 kDa. **F**) The indicated amounts of control and two independent Itgβ5-KD MDCK cell lysates were loaded for SDS-PAGE followed by detection of β5-integrins by western blotting with sheep anti-β5-integrin antibodies. The antibody recognized a band at ∼100 kDa that was down-regulated in one of the two Itgβ5-KD cell lysates. **G**) αV-integrins do not regulate the composition of β1-integrin heterodimers. Control, Itgα2- and ItgαV-KD#2 MDCK cell lines were metabolically labeled and β1-integrins precipitated as in C). The pattern of β1-integrins precipitated from control and ItgαV-KD cells is essentially identical. **H**) αV-integrins do not co-cluster with β1-integrins on Col I substrate. MDCK cells stably transfected with αV-integrin-RFP fusion protein were trypsinized and seeded onto FN (upper panel) or Col I (lower panel)-coated glass in the absence of serum and allowed to settle for 30 minutes. The cells were imaged using spinning disk confocal microscope and 63x oil-immersion objective. Localization of αV-integrin-RFP at the basal membrane is shown. Only relatively low-expressing cells were found but most of them showed clear accumulation of αV-integrin-RFP fusion protein into pericellular foci on FN whereas on Col I substrate only uniform basal staining was observed.
(TIF)

Figure S2 **αVβ6 integrin is the major adhesive FN receptor in MDCK cells.** *Cell adhesion analysis by cell washing assay:* Single cell suspensions of control and the indicated Itg-KD MDCK cells were allowed to settle for 90 minutes on **A**) fibronectin-, **B**) basement membrane-extract (BME)-, **C**) collagen I- or **D**) laminin-511 (LN-511)-coated tissue culture wells. Non-adherent cells were washed away and remaining adherent cells were fixed, stained and quantified. Adhesion of control cells to each coating was set to 1 and adhesion of the different Itg-KD cells is shown relative to the control. Each Itg-KD sample represents data from 4–10 independent experiments (shRNA#1 constructs) or 2–5 experiments (shRNA#2). Each value is normalized to a control value within the experiment and shows the mean + standard deviation (SD). P-values <0.01 are signified by (*) for constructs which were analyzed in at least 3 independent experiments. ND: not determined.
(TIF)

Figure S3 **Schemes of the SCFS setups.** **A**) *Measuring cell adhesion to collagen 1-coated supports:* The position of a laser beam (red line), that is reflected off the back of a calibrated AFM cantilever, on a photodiode (PD) measures the deflection of the cantilever and thus the force acting on the cantilever. A single cell is bound to an AFM cantilever *via* the lectin concanavalin A. It is lowered onto a collagen I-coated support until a contact force of ≈2 nN is recorded. After keeping the cell, at constant height, on the support for a preset contact time, it is retracted from the support until cell and substrate are completely separated. During the approach-retract cycle, the force acting on the cantilever is recorded and can be plotted in a force-distance (F–D) curve. During cantilever retraction, the maximum downward force acting on the cantilever is referred to as the maximum force needed to detach the cell from the substrate (F_D_). After the major detachment force peak, smaller unbinding events can be detected. The majority of these events correspond to the rupture of membrane nanotubes (tethers). Tethers (T) are characterized by long force plateaus of constant force. **B**) *Measuring mechanical maturation of FA using AFM:* Cells were allowed to grow for 12 hours on collagen I-coated petri dishes. A collagen I-coated bead, attached to the apex of a tipless AFM cantilever, is lowered onto the margin of an isolated cell until a force of 1 nN is applied. The bead is maintained at constant height for 2 minutes to facilitate strong binding between cell and bead. Subsequently the cell-attached bead is oscillated for 20 minutes with an amplitude of 200 nm and a frequency of 0.25 Hz. The oscillation curve at the bottom shows the oscillating piezo movement that oscillates the cantilever to which the bead is attached. During oscillation, the force acting on the cantilever is recorded and plotted in a force vs time curve (upper oscillation curve). The sections of the force-time curve that are recorded during upward movement of the cantilever are analyzed for maximum force difference, as displayed in the inset (upper oscillation curve).
(TIF)

Figure S4 **Depletion of αV-integrins in leads to a cell spreading defect in MK3 cells.** A) Control and ItgαV-KD MK3 cells were trypsinized, seeded onto Col I-coated coverslips and allowed to settle for 240 minutes. Cells were fixed and stained for actin (TRITC-Phalloidin, red) and nuclei (DAPI, blue). B) Quantitation of the data shows the mean cell spreading area (µm^2^/cell)+SD of 2 independent experiments performed in duplicates. For each experiment and coating condition ∼60–150 cells from 10–30 frames were analyzed. P-values <0.01 are signified by (*).
(TIF)

Table S1 **Validation of integrin-KD constructs.** **A**) shRNA target sequences used in this study and their respective KD-efficiencies. **B**) Oligos used for quantitative real-time PCR.
(DOC)

Movie S1 **Spreading dynamics of control, Itgβ1- and ItgαV-KD MDCK cells seeded onto collagen I substrate.** Control, Itgβ1- and ItgαV-KD MDCK cells stably expressing vinculin-GFP were trypsinized, seeded onto collagen I-coated glass-bottom tissue culture dishes and imaged with a spinning disk confocal microscope and 25x objective for indicated times. Note that some Itgβ1-KD cells are moving on the substrate indicating poor adhesion whereas most of the ItgαV-KD cells appear immobilized despite their inability to spread properly.
(AVI)

Movie S2 **Timelapse imaging of GFP-Tln1 fusion protein in control, ItgαV- and Itgβ1-KD MDCK cells seeded onto collagen I substrate.** Control, Itgβ1- and ItgαV-KD MDCK cells stably expressing talin-1-GFP were trypsinized, seeded onto collagen I-coated glass-bottom tissue culture dishes in serum-free cell culture medium, allowed to settle for 30–60 minutes and imaged for 4.5 hours with 3 minutes time intervals using TIRF-microscopy and a 63x oil-immersion objective.
(AVI)

Movie S3 **Timelapse imaging of GFP-vinculin fusion protein in control, ItgαV- and Itgβ1-KD MDCK cells seeded onto collagen I substrate.** Control, Itgβ1- and ItgαV-KD MDCK cells stably expressing vinculin-GFP were trypsinized, seeded onto collagen I-coated glass-bottom tissue culture dishes in serum-free cell culture medium, allowed to settle for 30–60 minutes and imaged for 4.5 hours with 3 minutes time intervals using TIRF-microscopy and a 63x oil-immersion objective.
(AVI)
