# Supplementary figures and images for: αV-Integrins Are Required for Mechanotransduction in MDCK Epithelial Cells
Source: PLoS One. 2013 Aug 19;8(8):e71485. doi: 10.1371/journal.pone.0071485 (PMC3747215; doi:10.1371/journal.pone.0071485)

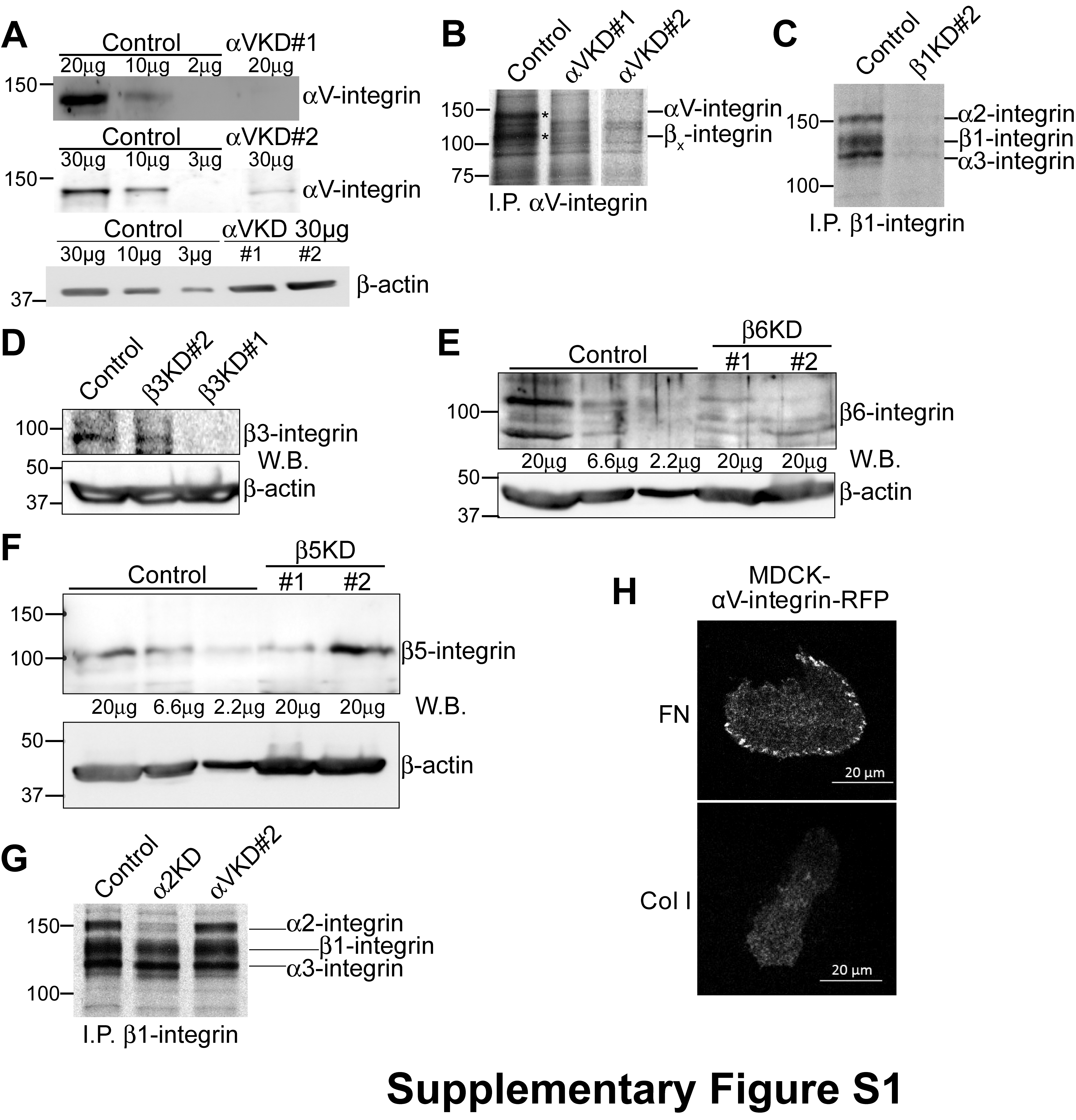

Supplement: Figure S1 — Analysis of integrin protein expression in the different integrin-KD cell lines. A) The indicated amounts of control and ItgαV-KD#1 and #2 MDCK cell lysates were loaded for SDS-PAGE followed by detection of αV-integrins by western blotting with rabbit anti-αV-integrin antibodies. β-actin was blotted as a loading control. B) Control MDCK and two independent ItgαV-KD cell lines were grown for 18 hours on Ø60 mm TC-plastic dishes and metabolically labeled for 18 hours with 35S-Methionine/Cysteine. αV-integrins were immunoprecipitated with rabbit polyclonal anti-αV-integrin antibodies as described in materials and methods. C) Control and Itgβ1-KD#2 MDCK cell lines were grown and metabolically labeled as in B) followed by immunoprecipitation of β1-integrins using rabbit polyclonal anti β1-integrin antibodies. The identity of the protein bands was confirmed with a series of metabolic labeling experiments using Itgα2- (Fig. S1G) and Itgα3-KD (data not shown) cells in which the respective protein bands were significantly reduced. D) Twenty micrograms of control and two Itgβ3-KD MDCK cell lysates were loaded for SDS-PAGE followed by detection with mouse monoclonal αVβ3-integrin antibodies. Only a faint band at ∼95 kDa was observed in the control cell lysate but the intensity of this band was further reduced in Itgβ3-KD#2 cells and it was undetectable in Itgβ3-KD#1 cell lysates E) The indicated amounts of control and two independent Itgαβ6-KD MDCK cell lysates were loaded for SDS-PAGE followed by detection of β6-integrins by western blotting with rabbit anti-β6-integrin antibodies. The antibody recognized two bands (∼110 kDa and ∼85 kDa) both of which appeared to be reduced in Itgβ6-KD cell lines. The calculated molecular weight of canine β6-integrin is 86 kDa. F) The indicated amounts of control and two independent Itgβ5-KD MDCK cell lysates were loaded for SDS-PAGE followed by detection of β5-integrins by western blotting with sheep anti-β5-integrin antibodies. The antibody [file pone.0071485.s001.tif]

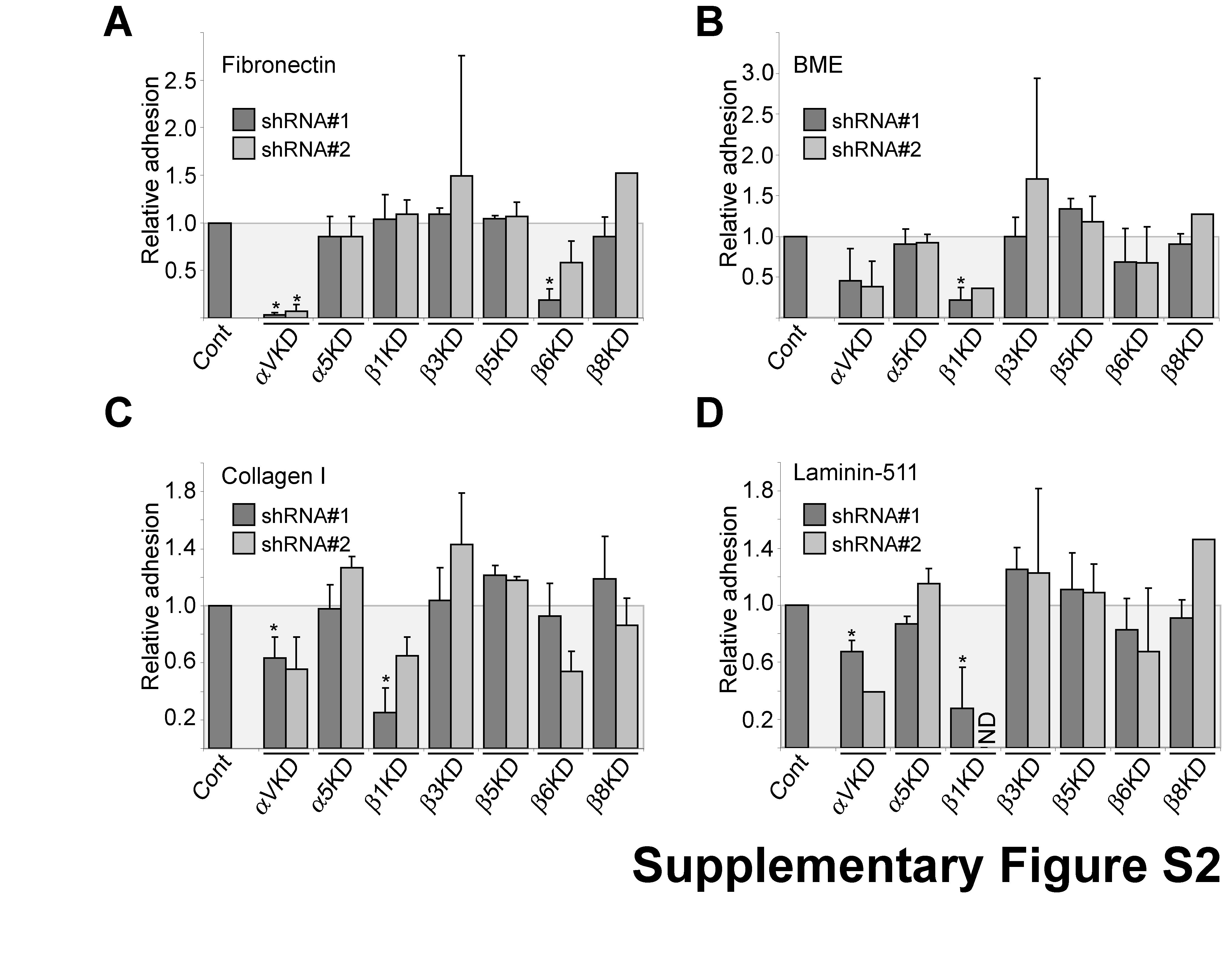

Supplement: Figure S2 — αVβ6 integrin is the major adhesive FN receptor in MDCK cells. Cell adhesion analysis by cell washing assay: Single cell suspensions of control and the indicated Itg-KD MDCK cells were allowed to settle for 90 minutes on A) fibronectin-, B) basement membrane-extract (BME)-, C) collagen I- or D) laminin-511 (LN-511)-coated tissue culture wells. Non-adherent cells were washed away and remaining adherent cells were fixed, stained and quantified. Adhesion of control cells to each coating was set to 1 and adhesion of the different Itg-KD cells is shown relative to the control. Each Itg-KD sample represents data from 4–10 independent experiments (shRNA#1 constructs) or 2–5 experiments (shRNA#2). Each value is normalized to a control value within the experiment and shows the mean + standard deviation (SD). P-values <0.01 are signified by (*) for constructs which were analyzed in at least 3 independent experiments. ND: not determined. (TIF) [file pone.0071485.s002.tif]

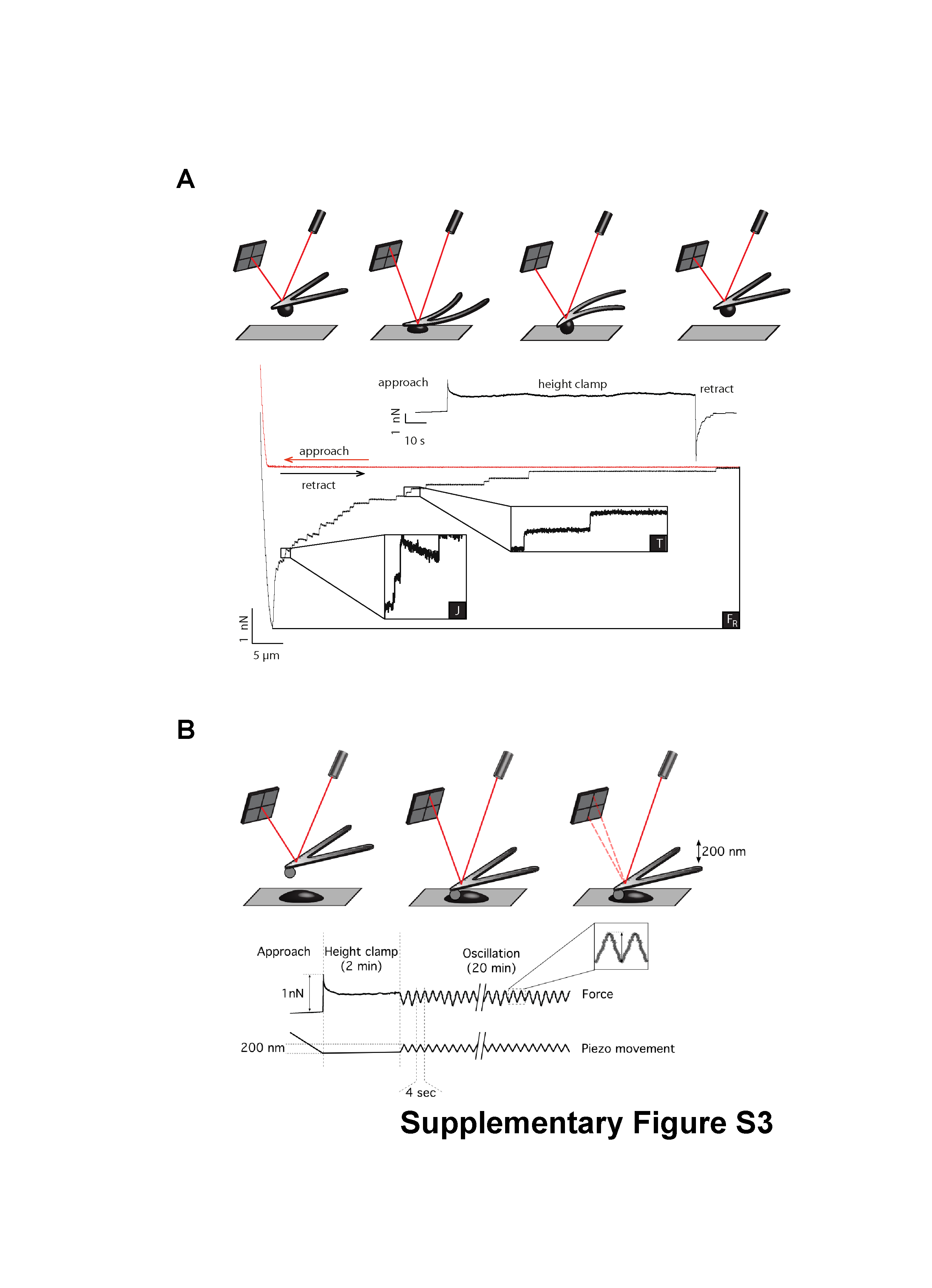

Supplement: Figure S3 — Schemes of the SCFS setups. A) Measuring cell adhesion to collagen 1-coated supports: The position of a laser beam (red line), that is reflected off the back of a calibrated AFM cantilever, on a photodiode (PD) measures the deflection of the cantilever and thus the force acting on the cantilever. A single cell is bound to an AFM cantilever via the lectin concanavalin A. It is lowered onto a collagen I-coated support until a contact force of ≈2 nN is recorded. After keeping the cell, at constant height, on the support for a preset contact time, it is retracted from the support until cell and substrate are completely separated. During the approach-retract cycle, the force acting on the cantilever is recorded and can be plotted in a force-distance (F–D) curve. During cantilever retraction, the maximum downward force acting on the cantilever is referred to as the maximum force needed to detach the cell from the substrate (FD). After the major detachment force peak, smaller unbinding events can be detected. The majority of these events correspond to the rupture of membrane nanotubes (tethers). Tethers (T) are characterized by long force plateaus of constant force. B) Measuring mechanical maturation of FA using AFM: Cells were allowed to grow for 12 hours on collagen I-coated petri dishes. A collagen I-coated bead, attached to the apex of a tipless AFM cantilever, is lowered onto the margin of an isolated cell until a force of 1 nN is applied. The bead is maintained at constant height for 2 minutes to facilitate strong binding between cell and bead. Subsequently the cell-attached bead is oscillated for 20 minutes with an amplitude of 200 nm and a frequency of 0.25 Hz. The oscillation curve at the bottom shows the oscillating piezo movement that oscillates the cantilever to which the bead is attached. During oscillation, the force acting on the cantilever is recorded and plotted in a force vs time curve (upper oscillation curve). The sections of the force-time curve that a [file pone.0071485.s003.tif]

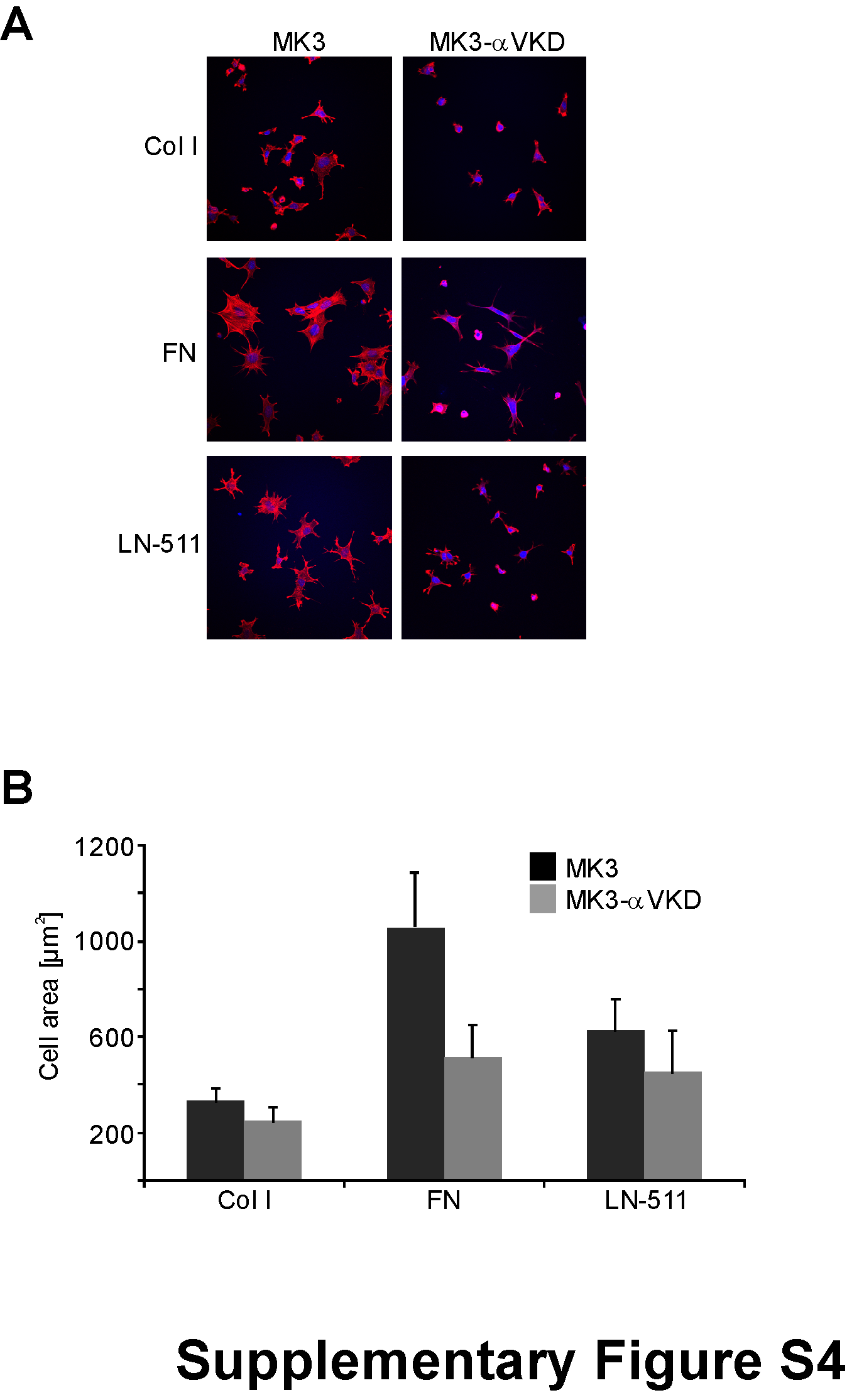

Supplement: Figure S4 — Depletion of αV-integrins in leads to a cell spreading defect in MK3 cells. A) Control and ItgαV-KD MK3 cells were trypsinized, seeded onto Col I-coated coverslips and allowed to settle for 240 minutes. Cells were fixed and stained for actin (TRITC-Phalloidin, red) and nuclei (DAPI, blue). B) Quantitation of the data shows the mean cell spreading area (µm2/cell)+SD of 2 independent experiments performed in duplicates. For each experiment and coating condition ∼60–150 cells from 10–30 frames were analyzed. P-values <0.01 are signified by (*). (TIF) [file pone.0071485.s004.tif]
