# Supplementary material for: αV-Integrins Are Required for Mechanotransduction in MDCK Epithelial Cells
Source: PLoS One. 2013 Aug 19;8(8):e71485. doi: 10.1371/journal.pone.0071485 (PMC3747215; doi:10.1371/journal.pone.0071485)
Supplement: Table S1 — Validation of integrin-KD constructs. A) shRNA target sequences used in this study and their respective KD-efficiencies. B) Oligos used for quantitative real-time PCR. (DOC) [file pone.0071485.s005.doc]

**Supplementary Table S1**

**A) shRNA target sequences used in this study and their respective KD-efficiencies**

| **Construct** | **Target sequence** | **Position in the target mRNA** | **KD-eff. [%]** |
| --- | --- | --- | --- |
| Itgα2-shRNA | GTGCAGATTACTCTCCTCAAA | 1121-1141 | 85 ± 9 |
| Itgα5- shRNA#1 | GGACCTCAGATCCTGAAATGT | 2979-2999 | 76 ± 16 |
| Itgα5- shRNA#2 | GGCCAGTCTTGCATTATCAGA | 1949-1969 | 67 ± 3 |
| ItgαV- shRNA#1 | GTGCCATCTTGTATGTGAAGT | 2579-2599 | 90 ± 5 |
| ItgαV- shRNA#2 | GACGAGCACTGTTTCTCCATA | 1427-1447 | 90 ± 2 |
| Itgβ1-shRNA#1 | GGGACGTGTTGGTAGACATT | 1548-1567 | 91 ± 3 |
| Itgβ1-shRNA#2 | GTGCAGAGCCTTCAATAAAG | 2013-2032 | 87 ± 11 |
| Itgβ3- shRNA#1 | GGCCAGATGATTCAAAGAATT | 344-364 | 90 ± 5 |
| Itgβ3- shRNA#2 | GCCTCCACTACCATGGATTAT | 913-933 | 58 ± 13 |
| Itgβ5- shRNA#1 | GTGTCATGATGTTCACCTACA | 2129-2149 | 94 ± 1 |
| Itgβ5- shRNA#2 | GGTTCGACAGGTGGAGGATTA | 444-464 | 73 ± 13 |
| Itgβ6- shRNA#1 | GTGGACTTGTATTACCTCATG | 604-624 | 86 ± 12 |
| Itgβ6- shRNA#2 | GCAACTGTGACTGTCAGAAAG | 1562-1582 | 71 ± 7 |
| Itgβ8- shRNA#1 | GGCTGTTCGACTGATTCAATA | 277-297 | 76 ± 2 |
| Itgβ8- shRNA#2 | CAGCTGTCTGTGAGAGTCATA | 785-805 | 74 ± 8 |
| Talin1-shRNA#1 | GTCCTCCAGCAGCAGTATAA | 129-150 | 89±9 |
| Talin1-shRNA#2 | GAGGCAACCACAGAACACATA | 6439-6460 | 80±8 |
| FAK-shRNA#1 | GGGTCAAGCTGGATTATTTCA | 787-807 | 81 ± 7 |
| FAK-shRNA#2 | GCCAACTCTGAATTTCTTCTA | 423-452 | 70 ± 2 |
| ILK-shRNA#1 | GGCGAAGCTCAATGAGAATCA | 588-609 | 85±10 |
| ILK-shRNA#2 | GACGTCAAGTTCTCCTTCCAA | 1015-1036 | 80±13 |

**B) oligos used for quantitative real-time PCR**

| **Target** | **Forward primer** | **Reverse primer** |
| --- | --- | --- |
| α5-integrin | GACGACACGGAGGACTTTGT | TGTCTGAGCCATTGAGGATG |
| αV-integrin | TCCAGGTGGAGCTTCTTTTG | TTCTTAGAGTGACCTGGAGACC |
| β1-integrin | ATCCCAGAGGCTCCAAAGAT | GCTGGAGCTTCTCTGCTGTT |
| β3-integrin | GACCTTTGAGTGTGGGGTGT | TCTTCCGAGCATTCACACTG |
| β5-integrin | AAGCCCATCTCCACACACTC | AGGAGAAGGGGCTCTCAGTC |
| β6-integrin | TGAGACCAGGCAGTGAACAG | CCGAGAGGTCCATGAGGTAA |
| β8-integrin | CGTGACTTCCGTCTTGGATT | CCTTTCTGGGTGGATGCTAA |
| Ubiquitin | TCCAAGACAAGGAGGGCATC | TTCTAGCTGTTTGCCCGCA |
| Talin1 | CCAGAAGGTTCCTTTGTGGA | GGCTGGTGTTTGACTTGGTT |
| FAK | AAGTGTGCTCTGGGGTCAAG | AGCCTTTGTCCGTGAGGTAA |
| ILK | AGCTCAACTTTCTGGCGAAG | CTTCACGACGATGTCATTGC |
